# A world renowned psychophysiologist: Kaoliang Chow

**DOI:** 10.1007/s13238-020-00797-5

**Published:** 2020-10-15

**Authors:** Lei Zhang, Lijun Wang, Benyu Guo, Yanyan Qian, Qingming Liu

**Affiliations:** 1 School of Psychology, Nanjing Normal University, Nanjing, 210046 China; 2 School of Educational Science, Anhui Normal University, Wuhu, 241000 China; 3 Social and Behavioral Sciences Facility, Leiden University, Leiden, 2300 RB Netherlands

**Figure 1:**
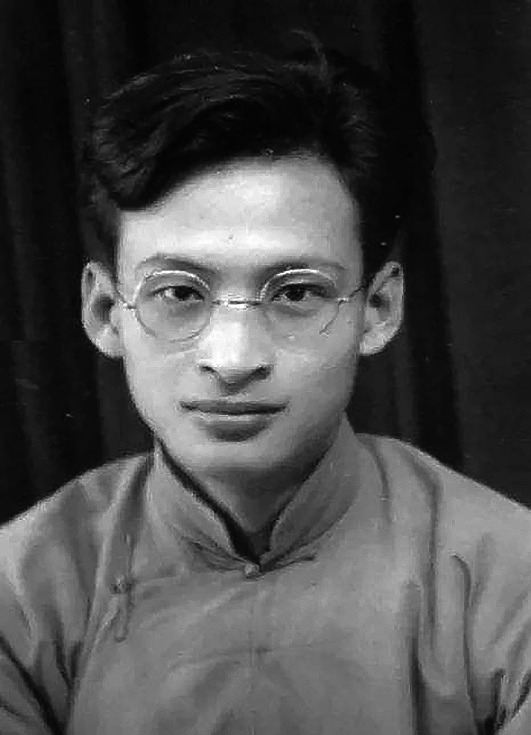
Kaoliang Chow (1918–1998)

Kaoliang Chow (周杲良, 1918–1998) (Fig. 1), was a world-renowned psychophysiologist and neurophysiologist (Meng, 2012). He had a prominent family background, was well educated, and made significant contributions towards understanding the relationship between brain and behavior, especially regarding the processing of vision by the brain. He was among the founders of the neuroscience doctoral program and a major figure in research and training at the Stanford University School of Medicine.

**Figure 2:**
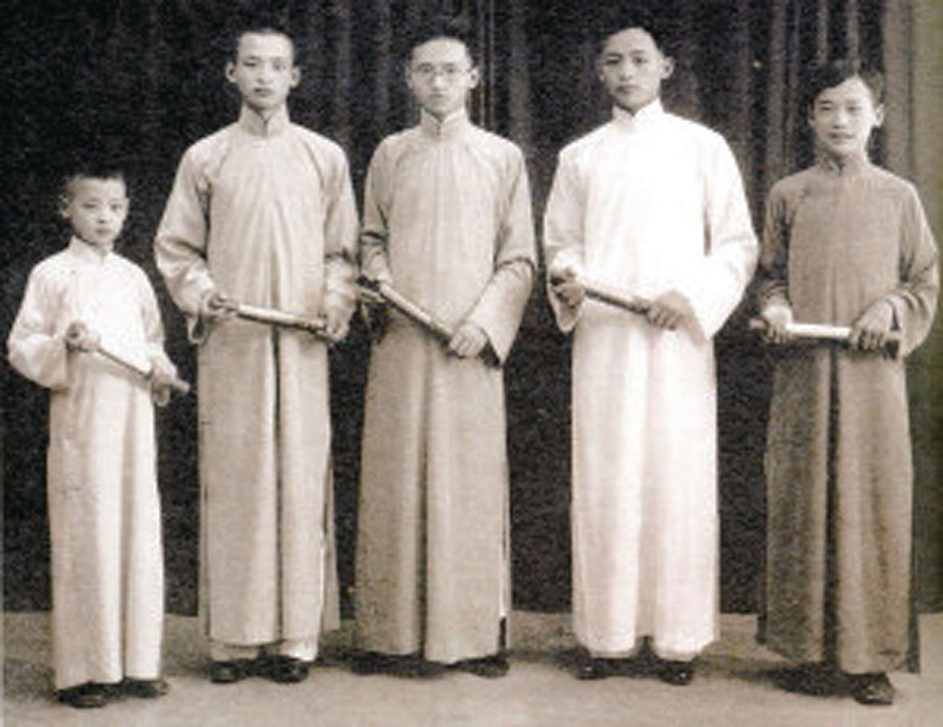
Kaoliang Chow (the second from left) with his brothers holding their diploma in 1935

**Figure 3:**
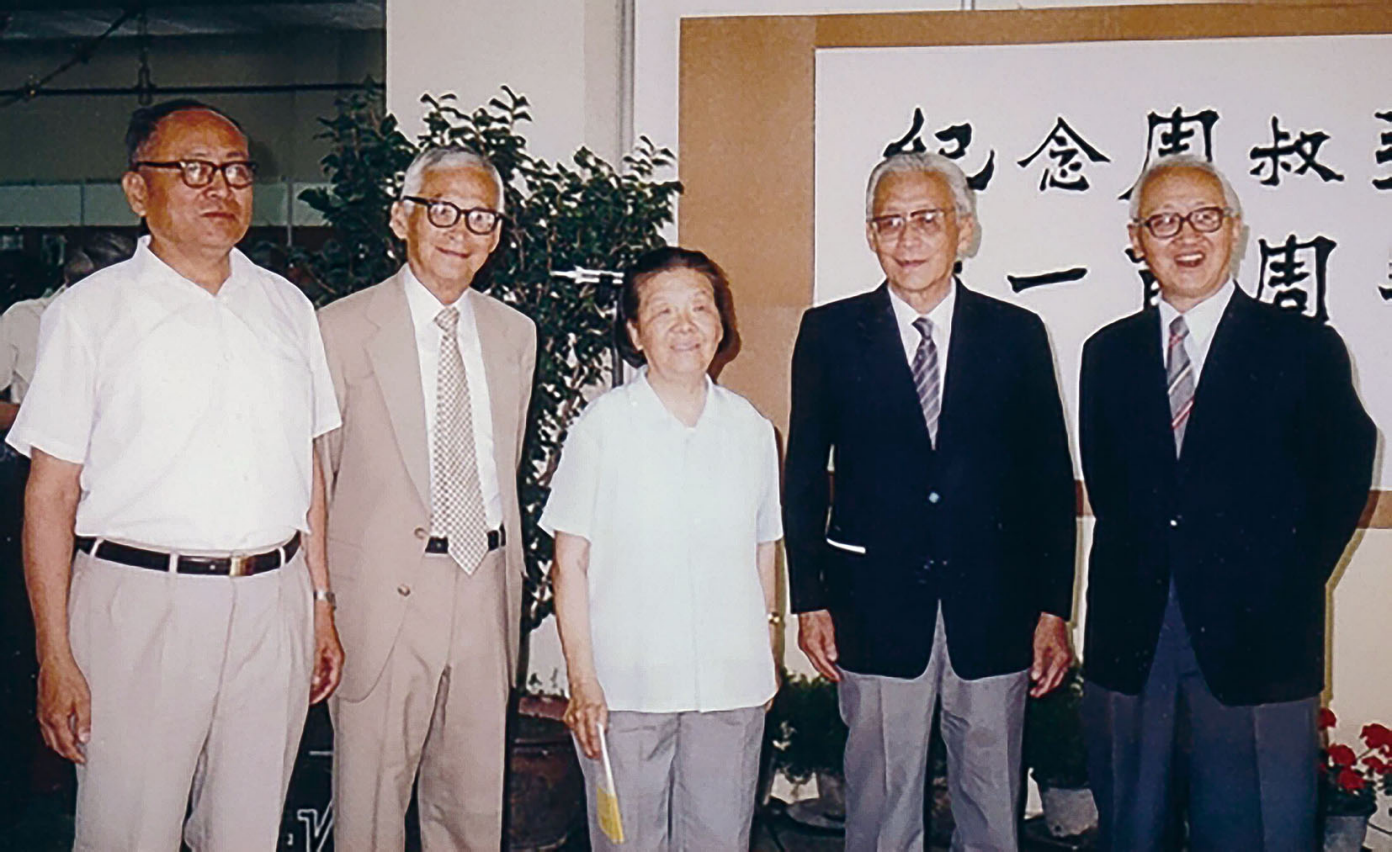
On June 25, 1991, Kaoliang Chow (the second from right) in the exhibition to commemorate the 100th anniversary of his father’s birth

On April 21, 1918, Chow was born in Tianjin, China, while his ancestral home was in Jiande county (currently Dongzhi) in the Anhui province of China. His family was well known and well-respected in China. His great-grandfather, Fu Chow (周馥), was a powerful government officer of the late Qing Dynasty and had managed several provinces, and his granduncle, Xuexi Chow (周学熙), served as the chief financial officer of the Beiyang government and was the forerunner of the modern industry and modern education. His father, Shutao Chow (周叔弢), the former vice-chairman of the Chinese People’s Political Consultative Conference (CPPCC), was a politician, a collector as well as a specialist on relics, and his uncle, Shujia Chow (周叔伽), was a Buddhist scholar and educator. Chow’s brothers are all famous scholars in different fields (Fig. 2). His prestigious family has had a great influence on academia. The famous contemporary writer, Huang Shang (黄裳), mentioned in the preface of Ruchang Chow’s (周汝昌) book Xian Qin Ji (献芹集) that Chow once brought the Chow’s block-printed edition of Qu Yuan Fu Zhu (屈原赋注) to school, and he introduced it as the first primer on science of edition (Chow, 1985). Raised in a literary family, Chow was addicted to reading, and he often wrote to his father that he did not have enough books to read (Chow, 2013). Being well-read, Chow had a solid foundation in different areas. Whether it was politics, economy, history, literature, or art, he could cite the classics and present his ideas with depth and dimensions (Yenching University Research Institute, 2002) (Fig. 3).

From 1938 to 1950, Chow studied at the Yenching University, followed by Harvard University, where he was deeply influenced by the top scholars from both China and America in psychology, neurobiology, and neuropsychology. In 1938, Chow began his college life at the Yenching University and graduated in 1943 (Zhang, Wang, and Qian, 1999). His teachers, including Zhiwei Lu (陆志伟), and Rende Xia (夏仁德) were all pioneers in the field of psychology in China. Chow studied psychology under their guidance, laying a sound foundation for future research. After graduation, Chow worked for 3 years at the Institute of Psychology, Academia Sinica. Next, he went to Harvard University for further studies and received his doctorate in 1950 (Zhang, Wang, and Qian, 1999). He did his doctoral studies under the supervision of Karl Lashley, a foremost physiological psychologist, and he was also sponsored by Gordon Allport, a social psychologist who was under the impression that Chow’s interest lay in psychology (Glickstein, 1999). In 1954, Chow was appointed as faculty at the Department of Physiology, University of Chicago, until Stanford hired him in 1961. He developed an excellent reputation following his contributions in organizing the neuroscience doctoral program and other academic projects. In 1983, he retired as an Emeritus professor (Yenching University Research Institute, 2002).

Chow’s research interest included: the physiology of the brain’s visual system, the neural basis of learning and memory, and the structure of the brain and thalamus. His initial studies had revealed that the striate cortex did not solely mediate vision, and he wanted to explore the other factors that influenced the visual system. Further investigation by Chow and his colleagues found that the striate cortex did not have a decisive effect on the visual system. They revealed that the visual system in monkeys (*Macaca mulatta*) was also influenced by the associative areas that were “a part of the cerebral cortex to which no definite motor or sensory function can be ascribed, and their neurons are generally thought to compose internuncial pools between afferent and efferent systems” (Chow, Blum, and Blum, 1951). They found that the discriminative capacities of the vision, somesthesia, and audition were partially affected by the ablations of the associative areas (Chow, Blum, and Blum, 1951). Subsequently, Chow and his colleagues demonstrated that in monkeys, visual discrimination was affected by the ablation of associative areas or temporal neocortex (Chow, 1952a, b), but visual attentiveness was not disturbed by temporal neocortical lesions (Chow and Orbach, 1957). Furthermore, Chow also explored the influence of the different areas of the cortex under various conditions, such as light, electric shock, and visual deprivation (Chow, 1955; Orbach and Chow, 1959; Chow and Stewart, 1972). For example, in his paper titled “Failure to demonstrate changes in the visual system of monkeys kept in darkness or in colored lights”, Chow discovered that “there were no detectable histological changes in the retina, superior colliculus, lateral geniculate, and striate area of these experimental animals.” under the different light conditions (Chow, 1955). Chow and his colleagues also demonstrated that the neocortical ablations in experimental animals (immature rhesus monkeys) had an insignificant effect on the overlearned visual discrimination, and these animals could retain visual discrimination when given additional training after satisfying the criterion (Chow and Survis, 1958). This series of studies on the visual system made him a major figure in this field, and one of his students, Mitchell Glichstein, an emeritus professor at the University of London, acknowledged that Chow’s research was “a milestone in opening up the field for the behavioral, anatomical, and physiological study of processing vision beyond the striate cortex.” (Glickstein, 1999).

Chow contributed substantially to this field through his experiments on the relationship between the brain and behavior. His studies revealed that there existed some connections among the bilateral temporal neocortical and pattern discrimination, visual object discrimination, and color discrimination (Chow, 1954; Chow and Survis, 1958); excessive training played a vital role in discernment recovery (Chow and Survis, 1958); the functional equivalence of both the eyes of a chimpanzee’s cub was not solely determined by the structure of the central visual system (Chow and Nissen, 1955). Chow and his colleague J. Orbach also studied the relationship between somatic area I and somatic area II and found that “the integrity of somatic area II could not compensate for a loss of somatic area I” (Orbach and Chow, 1959). This study plays a leading role in the field as it “represents a preliminary attempt to examine the effects of resections of somatic areas I and II, combined and separately, upon somesthetic discrimination in monkeys” (Orbach and Chow, 1959). In 1972, Chow investigated the conditioned avoidance response in crayfish and showed that there were “two parallel neural systems, one connected to the eyes and the other to the tail photoreceptor” (Chow and Leiman, 1972). The results of this study were consistent with previous reports, which indicated that the tail photoreceptor and the eyes were “functionally equivalent in transmitting sensory information to a common center concerned with learning processes” (Chow and Leiman, 1972).

Additionally, Chow’s work described the structure of the cerebral cortex and thalamic cortex, the relationship between the cerebrum and thalamus, and the role of the cerebral cortex in epilepsy. He primarily explored the cellular characteristics in the cerebral cortex and thalamic cortex, the projection relationship of the cerebral cortex and thalamic cortex, and the callosal projections from the striate cortex. He discovered the average number of the cells in the lateral geniculate and the striate cortex of *Macaca mulatta* (Chow, Blum, and Blum, 1950), and the average number of cells/0.002 mm^2^ in the auditory central nervous system of the brains of two monkeys (Chow, 1951). He also found that “after extensive cortical destruction involving several cortical fields, the reticular nucleus shows extensive cell-atrophy, which appears to be a summation of separate regional reticular degenerations” (Chow, 1950, 1952c). Subsequently, Chow and his coagent Karl H. Pribram described in detail the projection relationship between the thalamic ventrolateral nuclear group and cerebrum (Chow and Pribram, 1956). In a study on the callosal projections from the striate cortex, Chow et al. found that there was a difference in callosal distributions of young rabbits and adult rabbits, indicating that there could be callosal efferent fibers originating from the medial striate cortex in the rabbit pups (Chow, Baumbach, and Glanzman, 1978). This hypothesis was confirmed by the following study published in Experimental Brain Research, “Callosal projections of the striate cortex in the neonatal rabbit,” where they found that the callosal projections of newborn rabbits originated from cells in the medial striate cortex (Chow, Baumbach, and Lawson, 1981). These findings were of great significance, as he said: “The present findings together with those cited above (other similar studies), point to the dynamic nature of the developmental process in establishing neuronal connections.” (Chow, Baumbach, and Lawson, 1981). It is evident that Chow had established a leading position in the field of brain function research based on his profound theoretical foundation and abundant research. Moreover, Chow’s research paradigm and authority were highly recognized by Bingxuan Xu (徐秉烜), a Chinese neurobiologist, who repeatedly quoted Chow’s research in his reviews on the progress of the neural basis and physiological mechanism of memory (Xu, 1961, 1965).

Thus, Chow was a Chinese American who was born, raised, and educated in China. He was one of the best scholars in his field and made a substantial contribution to physiological psychology. His team established that visual processing was not limited to the striate cortex. As an international authority in the field of neurophysiology, Chow actively participated in the establishment of the Stanford University’s psychophysiology program and the research work at the School of Medicine, which laid the foundation for the School of Medicine at Stanford University to become one of the world’s top medical schools. Chow’s name appeared in Who’s Who and American Men and Women of Science in the USA (Zhang, 1994). He was also mentioned as a distinguished member of the Chow family in Chinese books (Wang, 2007). Moreover, he was very concerned about the educational, academic, and construction situation in China, and had returned to China several times to give lectures (Yenching University Research Institute, 2002). Chow’s achievements were rooted in his family environment and the learning spirit of the two Alma Maters: Yenching University and Harvard University; as a perfect combination of Chinese and Western culture.
