# Correction to: A world renowned psychophysiologist: Kaoliang Chow

**DOI:** 10.1007/s13238-021-00886-z

**Published:** 2021-11-03

**Authors:** Lei Zhang, Lijun Wang, Benyu Guo, Yanyan Qian, Qingming Liu

**Affiliations:** 1 School of Psychology, Nanjing Normal University, Nanjing, 210046 China; 2 School of Educational Science, Anhui Normal University, Wuhu, 241000 China; 3 Social and Behavioral Sciences Facility, Leiden University, Leiden, 2300 RB Netherlands

## Correction to: Protein Cell 10.1007/s13238-020-00797-5

In the Figure 3 legend, “the second from right” should be revised as “the second from left”.

